# The impact of epistasis in the heterosis and combining ability analyses

**DOI:** 10.3389/fpls.2023.1168419

**Published:** 2023-04-18

**Authors:** José Marcelo Soriano Viana

**Affiliations:** Department of General Biology, Federal University of Viçosa, Viçosa, MG, Brazil

**Keywords:** epistasis, linkage disequilibrium, heterosis, combining ability, diallel

## Abstract

The current theoretical knowledge concerning the influence of epistasis on heterosis is based on a simplified multiplicative model. The objective of this study was to assess how epistasis affects the heterosis and combining ability analyses, assuming additive model, hundreds of genes, linkage disequilibrium (LD), dominance, and seven types of digenic epistasis. We developed the quantitative genetics theory for supporting the simulation of the individual genotypic values in nine populations, the selfed populations, the 36 interpopulation crosses, 180 doubled haploids (DHs), and their 16,110 crosses, assuming 400 genes on 10 chromosomes of 200 cM. Epistasis only affects population heterosis if there is LD. Only additive × additive and dominance × dominance epistasis can affect the components of the heterosis and combining ability analyses of populations. Epistasis can have a negative impact on the heterosis and combining ability analysis of populations, leading to wrong inferences regarding the identification of superior and most divergent populations. However, this depends on the type of epistasis, percentage of epistatic genes, and magnitude of their effects. Except for duplicate genes with cumulative effects and non-epistatic genic interaction, there was a decrease in the average heterosis by increasing the percentage of epistatic genes and the magnitude of their effects. The same results are generally true for the combining ability analysis of DHs. The combining ability analyses of subsets of 20 DHs showed no significant average impact of epistasis on the identification of the most divergent ones, regardless of the number of epistatic genes and magnitude of their effects. However, a negative effect on the assessment of the superior DHs can occur assuming 100% of epistatic genes, but depending on the epistasis type and the epistatic effect magnitude.

## Introduction

The knowledge on the molecular basis of heterosis is increasing from studies involving metabolomic-, proteomic-, transcriptomic-, and genomic-based analyses (Shi et al., 2019; Yi et al., 2019; Li et al., 2020; Liu et al., 2020a; Luo et al., 2021). The results from these studies—differentially accumulated metabolites and proteins and differentially expressed genes in the inbred lines and the single cross, as well as heterotic and epistatic candidate genes from genome-wide association studies (GWAS) and quantitative trait loci (QTL) mapping—have provided consistent evidence supporting the main hypotheses that explain the genetic basis of heterosis: dominance complementation, overdominance, and epistasis (Kaeppler, 2012; Schnable and Springer, 2013; Liu et al., 2020b; Mackay et al., 2021). In these reviews, the authors emphasize that the hypotheses are non-mutually exclusive, that no simple unifying explanation for heterosis exists, and that, because heterosis is of greatest magnitude for highly complex traits, it should be attributable to a large number of genes with small effects showing intra- and inter-allelic interaction, most of these genes showing dominance.

The planned use of heterosis has revolutionized maize breeding since the 1930s and is also currently employed in modern rice and tomato breeding. From the quantitative genetics point of view, assuming absence of epistasis, the heterosis between populations is a function of dominance and squared difference of allelic frequencies (Gardner and Eberhart, 1966). The most widely used method for heterosis analysis (analysis II) was proposed by Gardner and Eberhart (1966). However, the most employed methods for the analysis of diallel crosses for cross- and self-pollinated crops were proposed by Griffing (1956). Griffing’s experimental methods and models (random or fixed) can be summarized as combining ability analyses.

Regarding open-pollinated populations, analysis II of Gardner and Eberhart (1966) and experimental method 2, model 1 (fixed) of Griffing (1956) are equivalent. The variety effect in the restricted model, the variety mean in the unrestricted model (because the variety effect is not estimable), and the general combining ability (GCA) effect indicate the superiority of the population regarding allelic frequencies. If there is dominance, the heterosis/heterosis effect and the specific combining ability (SCA) effect express the differences of allelic frequencies between populations. The average heterosis and the predominant sign of the SCA effects of a population with itself indicate the dominance direction. The variety heterosis/variety heterosis effect and the absolute value of the SCA effect of a population with itself express the differences of allelic frequencies between the population and the average frequencies in the other diallel parents. The specific heterosis/specific heterosis effect jointly expresses the differences of allelic frequency between the populations and between the populations and the average frequencies in the parental group (Viana, 2000a; Viana, 2000b) (see also the erratum in Viana (2002)). By including the selfed populations, the change in the population mean due to inbreeding also indicates the dominance direction but additionally the populations with higher genetic variability (allelic frequencies closer to 0.5) (Viana and Matta, 2003).

Currently, most of the studies involving diallel crosses with populations and inbred/pure/doubled haploid (DH) lines are focused on the identification of heterotic groups, most of them including molecular markers (Laude and Carena, 2015; Lariepe et al., 2017; Punya et al., 2019; Yu et al., 2020). The main findings from these studies are that the suggested heterotic groups relate with previously known heterotic groups, geographical origin, and pedigree and that the correlation between heterosis or SCA effect with molecular divergence is not consistent. For maize grain yield, the correlation ranged from intermediate negative (−0.38) to intermediate positive (0.60).

Few previous theoretical studies prove the contribution of epistasis for heterosis. Assuming combined multiplicative action of two additive genes, Minvielle (1987) and Schnell and Cockerham (1992) concluded that dominance is not necessary for heterosis. Additionally, Schnell and Cockerham (1992) showed that the multiplicative action of more genes increases the contribution of dominance, but not epistasis, to heterosis. Cockerham and Zeng (1996) and Garcia et al. (2008) modeled epistatic linked QTLs. Their QTL mapping for maize and rice agronomic traits showed that the potential of additive × additive, additive × dominance, and dominance × dominance epistatic effects for linked QTLs can be very substantial. Because the current theoretical knowledge concerning the influence of epistasis on heterosis is based on a multiplicative model, assuming very few genes, only additive × additive epistasis, and linkage equilibrium, the objective of this simulation-based study was to assess the impact of epistasis in the heterosis and combining ability analyses, assuming additive model, hundreds of genes, linkage disequilibrium (LD), dominance, and seven types of digenic epistasis.

## Material and methods

### Theory

Assume N (N > 3) non-inbred random cross populations in the Hardy–Weinberg equilibrium, LD, and digenic epistasis. Based on the quantitative genetics theory for modeling epistasis and LD developed by Kempthorne (1954) and Kempthorne (1973), respectively, the genotypic mean of the jth population (generation 0) is


$$M_{j}=m+v_{j}+E{\left(AA\right)}_{j}^{\left(0\right)}+E{\left(DD\right)}_{j}^{\left(0\right)}=m+v_{j}^{*}$$

where m is the sum of the means of the genotypic values of the homozygotes for each gene, $\;v_{j}$ is the variety effect assuming no epistasis, $E{\left(AA\right)}_{j}^{\left(0\right)}$ is the expectation of the additive × additive epistatic genetic values of the individuals, $E{\left(DD\right)}_{j}^{\left(0\right)}$ is the expectation of the dominance × dominance epistatic genetic values, and $v_{j}^{*}$ is the variety effect. The parametric value of $v_{j}$ was derived by Viana (2000a) (see also the erratum in Viana (2002)). For two epistatic genes (A/a and B/b),


$$E{\left(AA\right)}_{j}^{\left(0\right)}=2\Delta_{abj}^{\left(-1\right)}\left(\alpha_{A}\alpha_{B}-\alpha_{A}\alpha_{b}-\alpha_{a}\alpha_{B}+\alpha_{a}\alpha_{b}\right)=2\Delta_{abj}^{\left(-1\right)}\left(aa\right)$$

$$E{\left(DD\right)}_{j}^{\left(0\right)}={\left[\Delta_{abj}^{\left(-1\right)}\right]}^{2}\left(\delta_{AA}\delta_{BB}-2\delta_{AA}\delta_{Bb}+\delta_{AA}\delta_{bb}-2\delta_{Aa}\delta_{BB}+4\delta_{Aa}\delta_{Bb}-2\delta_{Aa}\delta_{bb}+\delta_{aa}\delta_{BB}-2\delta_{aa}\delta_{Bb}+\delta_{aa}\delta_{bb}\right)={\left[\Delta_{abj}^{\left(-1\right)}\right]}^{2}\left(dd\right)$$

where $\Delta_{abj}^{\left(-1\right)}$ is the measure of LD in the gametic pool of the generation −1 (the difference between the products of the haplotypes, $\Delta_{abj}^{\left(-1\right)}\;=P_{ABj}^{\left(-1\right)}.P_{abj}^{\left(-1\right)}-P_{Abj}^{\left(-1\right)}.P_{aBj}^{\left(-1\right)}$ ) (Kempthorne, 1973), and αα and δδ stand for the additive × additive and dominance × dominance epistatic effects, respectively.

Because the population is not inbred and taking into account the restrictions proposed by Kempthorne (1954),


$$E{\left(AD\right)}_{j}^{\left(0\right)}=f_{22j}^{\left(0\right)}\left(2\alpha_{A}\delta_{BB}\right)+f_{21j}^{\left(0\right)}\left(2\alpha_{A}\delta_{Bb}\right)+\ldots +f_{00j}^{\left(0\right)}\left(2\alpha_{a}\delta_{bb}\right)=E{\left(DA\right)}_{j}^{\left(0\right)}=0$$

where $f_{ikj}^{\left(0\right)}$ is the probability of the genotype with i and k copies of the genes that increase the trait expression (A and B) (i, k = 0, 1, or 2). These probabilities are presented by Viana (2004), where, for example, $f_{22j}^{\left(0\right)}=p_{aj}^{2}p_{bj}^{2}+2p_{aj}p_{bj}\Delta_{abj}^{\left(-1\right)}+{\left[\Delta_{abj}^{\left(-1\right)}\right]}^{2}$ , where p stands for the frequency of the gene that increases the trait expression.

The genotypic mean of the interpopulation cross between the jth and the j′th populations is


$$M_{jj'}=m+\frac{1}{2}v_{j}+\frac{1}{2}v_{j'}+H_{jj'}+E{\left(AA\right)}_{jj'}+E{\left(DD\right)}_{jj'}=m+\frac{1}{2}v_{j}+\frac{1}{2}v_{j'}+H+H_{j}+H_{j'}+S_{jj'}+E{\left(AA\right)}_{jj'}+E{\left(DD\right)}_{jj'}$$

where $H_{jj'}$ , H , $H_{j}$ , and $S_{jj'}$ are, respectively, the heterosis, the average heterosis, the variety heterosis, and the specific heterosis assuming no epistasis, $E{\left(AA\right)}_{jj'}$ is the expectation of the additive × additive values in the F_1_, and $E{\left(DD\right)}_{jj'}$ is the expectation of the dominance x dominance values in the F_1_. The parametric values of the components $H_{jj'}$ , H , $H_{j}$ , and $S_{jj'}$ were derived by Viana (2000a). For two epistatic genes (see the derivation in the appendix),


$$E{\left(AA\right)}_{jj'}=\Delta_{abj}^{\left(0\right)}\left(aa\right)+\Delta_{abj'}^{\left(0\right)}\left(aa\right)=\left(1-r_{ab}\right)\left(E{\left(AA\right)}_{j}^{\left(0\right)}+E{\left(AA\right)}_{j'}^{\left(0\right)}\right)/2$$

$$E{\left(AD\right)}_{jj'}=E{\left(DA\right)}_{jj'}=0$$

$$E{\left(DD\right)}_{jj'}=\Delta_{abj}^{\left(0\right)}.\Delta_{abj'}^{\left(0\right)}\left(dd\right)={\left(1-r_{ab}\right)}^{2}\Delta_{abj}^{\left(-1\right)}.\Delta_{abj'}^{\left(-1\right)}\left(dd\right)$$

where $r_{ab}$ is the recombination frequency.

Then,


$$\begin{array}{l} {\text{M}}_{jj'}=\text{m}+\frac{1}{2}v_{j}^{*}+\frac{1}{2}v_{j'}^{*}+H_{jj'}+\left\{E{\left(AA\right)}_{jj'}-\left(1/2\right)\left[E{\left(AA\right)}_{j}^{\left(0\right)}+E{\left(AA\right)}_{j'}^{\left(0\right)}\right]\right\}\\ \;+\left\{E{\left(DD\right)}_{jj'}-\left(1/2\right)\left[E{\left(DD\right)}_{j}^{\left(0\right)}+E{\left(DD\right)}_{j'}^{\left(0\right)}\right]\right\}=\text{m}+\frac{1}{2}v_{j}^{*}+\frac{1}{2}v_{j'}^{*}+H_{jj'}^{*}\\ \;=\text{m}+\frac{1}{2}v_{j}^{*}+\frac{1}{2}v_{j'}^{*}+H^{*}+H_{j}^{*}+H_{j'}^{*}+S_{jj'}^{*} \end{array}$$

where


$$H^{*}=\left(1/C_{N}^{2}\right)\sum_{j=1<}^{N-1}\sum_{j^{\prime }=2}^{N}H_{jj'}^{*}=H+\left[E{\left(AA\right)}_{..}-E{\left(AA\right)}_{.}^{\left(0\right)}\right]+\left[E{\left(DD\right)}_{..}-E{\left(DD\right)}_{.}^{\left(0\right)}\right]$$

$$\begin{array}{l} H_{j}^{*}=\left[1/\left(N-1\right)\right]\sum_{\begin{array}{c} j'=1\\ j'\neq j \end{array}}^{N}H_{jj'}^{*}=H_{j}+\left\{E{\left(AA\right)}_{j.}-\left(1/2\right)\left[E{\left(AA\right)}_{j}^{\left(0\right)}+\left[1/\left(N-1\right)\right]\sum_{\begin{array}{c} j'=1\\ j'\neq j \end{array}}^{N}E{\left(AA\right)}_{j'}^{\left(0\right)}\right]\right\}\\ \;+\left\{E{\left(DD\right)}_{j.}-\left(1/2\right)\left[E{\left(DD\right)}_{j}^{\left(0\right)}+\left[1/\left(N-1\right)\right]\sum_{\begin{array}{c} j'=1\\ j'\neq j \end{array}}^{N}E{\left(DD\right)}_{j'}^{\left(0\right)}\right]\right\} \end{array}$$

and $S_{jj'}^{*}=H_{jj'}^{*}-H^{*}-H_{j}^{*}+H_{j'}^{*}$ .

Thus, assuming LD, only the additive × additive and dominance × dominance epistatic effects affect the variety effect and the heteroses. However, as demonstrated below, all epistatic effects affect the change in the population mean due to inbreeding. The genotypic mean of the jth selfed population is


$$M_{js}=m+v_{j}+d_{j}+E{\left(AA\right)}_{js}^{\left(n\right)}+E{\left(AD\right)}_{js}^{\left(n\right)}+E{\left(DA\right)}_{js}^{\left(n\right)}+E{\left(DD\right)}_{js}^{\left(n\right)}$$

where $d_{j}$ is the change in the population mean due to inbreeding assuming no epistasis and n is the number of selfing generations. The parametric value of $d_{j}$ was derived by Viana and Matta (2003). For two epistatic genes, the epistatic components are


$$E{\left(AA\right)}_{js}^{\left(n\right)}=E{\left(AA\right)}_{j}^{\left(0\right)}+c_{1}\left(1-2r_{ab}\right)\Delta_{abj}^{\left(-1\right)}\left(aa\right)$$

$$E{\left(AD\right)}_{js}^{\left(n\right)}=F\left(q_{bj}-p_{bj}\right)\Delta_{abj}^{\left(-1\right)}\left(\alpha_{A}\delta_{BB}-2\alpha_{A}\delta_{Bb}+\alpha_{A}\delta_{bb}-\alpha_{a}\delta_{BB}+2\alpha_{a}\delta_{Bb}-\alpha_{a}\delta_{bb}\right)+\left[c_{1}\left(1-2r_{ab}\right)\Delta_{abj}^{\left(-1\right)}/2\right]\left(\alpha_{A}\delta_{BB}-\alpha_{A}\delta_{bb}-\alpha_{a}\delta_{BB}+\alpha_{a}\delta_{bb}\right)$$

$$E{\left(DA\right)}_{js}^{\left(n\right)}=F\left(q_{aj}-p_{aj}\right)\Delta_{abj}^{\left(-1\right)}\left(\delta_{AA}\alpha_{B}-2\delta_{Aa}\alpha_{B}+\delta_{aa}\alpha_{B}-\delta_{AA}\alpha_{b}+2\delta_{Aa}\alpha_{b}-\delta_{aa}\alpha_{b}\right)+\left[c_{1}\left(1-2r_{ab}\right)\Delta_{abj}^{\left(-1\right)}/2\right]\left(\delta_{AA}\alpha_{B}-\delta_{aa}\alpha_{B}-\delta_{AA}\alpha_{b}+\delta_{aa}\alpha_{b}\right)$$

$$E{\left(DD\right)}_{js}^{\left(n\right)}=E{\left(DD\right)}_{j}^{\left(0\right)}+p_{1}\delta_{AA}\delta_{BB}+\ldots +p_{9}\delta_{aa}\delta_{bb}$$

where $c_{1}=2\left\{1-{\left[\left(1-2r_{ab}\right)/2\right]}^{n}\right\}/\left(1+2r_{ab}\right)$ , F is the inbreeding coefficient, αδ and δα stand for the additive × dominance and dominance × additive epistatic effects, and, for example, the probability $p_{1}$ is


$$p_{1}=\left(F/2\right)\left(f_{21j}^{\left(0\right)}+f_{12j}^{\left(0\right)}+f_{11j}^{\left(0\right)}/2\right)-\left(1-F\right)\left(1-c^{n}\right)f_{11j}^{\left(0\right)}/4+c_{1}\left(1-2r_{ab}\right)\Delta_{abj}^{\left(-1\right)}/4$$

where $c=1-2r_{ab}\left(1-r_{ab}\right)$ .

Then,


$$M_{js}=m+v_{j}^{*}+d_{j}^{*}$$

where $d_{j}^{*}=d_{j}+\left[E{\left(AA\right)}_{js}^{\left(n\right)}-E{\left(AA\right)}_{j}^{\left(0\right)}\right]+E{\left(AD\right)}_{js}^{\left(n\right)}+E{\left(DA\right)}_{js}^{\left(n\right)}+\left[E{\left(DD\right)}_{js}^{\left(n\right)}-E{\left(DD\right)}_{j}^{\left(0\right)}\right]$ .

Assuming no LD, $E{\left(AA\right)}_{js}^{\left(n\right)}=E{\left(AD\right)}_{js}^{\left(n\right)}=E{\left(DA\right)}_{js}^{\left(n\right)}=0$ . In the case of a combining ability analysis, the genotypic means of the jth population and the interpopulation cross between the jth and the j′th populations are, respectively,


$$M_{jj}=M_{..}+2g_{j}^{*}+s_{jj}^{*}$$

$$M_{jj'}=M_{..}+g_{j}^{*}+g_{j'}^{*}+s_{jj'}^{*}$$

where $M_{..}=\left(1/N^{2}\right)\sum_{j=1}^{N}\sum_{j^{\prime }=1}^{N}M_{jj^{\prime }}=\left(1/N\right)\sum_{j=1}^{N}M_{j.}$ is the diallel mean, $g_{j}^{*}$ is the GCA effect for population j, $s_{jj}^{*}$ is the SCA effect of a population with itself, and $s_{jj'}^{*}$ is the SCA effect for populations j and j′. The GCA effect is, assuming LD and epistasis,


$$g_{j}^{*}=M_{j.}-M_{..}=g_{j}+aa_{j.}^{*}+dd_{j.}^{*}$$

where $g_{j}$ is the GCA effect assuming no epistasis. The parametric value of $g_{j}$ was derived by Viana (2000b) (see also the erratum in Viana (2002)). The additive × additive and dominance × dominance epistatic components are


$$aa_{j.}^{*}=\left(1/N\right)\left[E{\left(AA\right)}_{j}^{\left(0\right)}+\sum_{\begin{array}{c} j^{\prime }=1\\ j'\neq j \end{array}}^{N}E{\left(AA\right)}_{jj^{\prime }}\right]-\left(1/N^{2}\right)\left[\sum_{j=1}^{N}E{\left(AA\right)}_{j}^{\left(0\right)}+2\sum_{j=1<}^{N-1}\sum_{j^{\prime }=2}^{N}E{\left(AA\right)}_{jj^{\prime }}\right]=aa_{j.}-aa_{..}$$

$$dd_{j.}^{*}=\left(1/N\right)\left[E{\left(DD\right)}_{j}^{\left(0\right)}+\sum_{\begin{array}{c} j^{\prime }=1\\ j'\neq j \end{array}}^{N}E{\left(DD\right)}_{jj^{\prime }}\right]-\left(1/N^{2}\right)\left[\sum_{j=1}^{N}E{\left(DD\right)}_{j}^{\left(0\right)}+2\sum_{j=1<}^{N-1}\sum_{j^{\prime }=2}^{N}E{\left(DD\right)}_{jj^{\prime }}\right]=dd_{j.}-dd_{..}$$

Note that $\sum_{j=1}^{N}g_{j}^{*}=0$ , for all j, because $\sum_{j=1}^{N}g_{j}=0$ , for all j (Viana, 2000b). The SCA effect of a population with itself is


$$s_{jj}^{*}=s_{jj}+E{\left(AA\right)}_{j}^{\left(0\right)}-aa_{..}-2aa_{j.}^{*}+E{\left(DD\right)}_{j}^{\left(0\right)}-dd_{..}-2dd_{j.}^{*}$$

where $s_{jj}$ is the SCA effect of a population with itself assuming no epistasis. The parametric value of ${\text{s}}_{\text{jj}}$ was derived by Viana (2000b). Finally, the SCA effect for the populations j and j′ is


$$s_{jj'}^{*}=s_{jj'}+E{\left(AA\right)}_{jj^{\prime }}-aa_{j.}^{*}-aa_{j^{\prime }.}^{*}-aa_{..}+E{\left(DD\right)}_{jj^{\prime }}-dd_{j.}^{*}-dd_{j^{\prime }.}^{*}-dd_{..}$$

where $s_{jj'}$ is the SCA effect for the populations j and j′ assuming no epistasis. The parametric value of $s_{jj'}$ was derived by Viana (2000b). Note that $\sum_{j'=1}^{N}s_{jj'}^{*}=0$ , for all j, because $\sum_{j'=1}^{N}s_{jj'}=0$ , for all j. Note also that $\sum_{j=1}^{N}s_{jj}^{*}+2\sum_{j=1<}^{N-1}\sum_{j^{\prime }=2}^{N}s_{jj'}^{*}=0$ because $\sum_{j=1}^{N}s_{jj}+2\sum_{j=1<}^{N-1}\sum_{j^{\prime }=2}^{N}s_{jj'=\;0}$ (Viana, 2000b). Thus, this combining ability model is restricted with N + 1 linearly independent restrictions (a full-rank model). The genotypic mean of the jth selfed population is $M_{jjs}=M_{..}+2g_{j}^{*}+s_{jj}^{*}+d_{j}^{*}$ .

In the case of a diallel involving N DH/inbred/pure lines, the genotypic value of a single cross is $M_{jj'}=M_{..}+g_{j}+g_{j'}+s_{jj'}+I_{jj^{\prime }}=M_{..}+g_{j}^{*}+g_{j'}^{*}+s_{jj'}^{*}$ , where $M_{..}=\left(1/C_{N}^{2}\right)\sum_{j=1<}^{N-1}\sum_{j^{\prime }=2}^{N}M_{jj'}$ is the diallel mean, $I_{jj'}$ is the epistatic genetic value, $g_{j}^{*}=\left(N-1\right)\sum_{j'=1}^{N\left(j'\neq j\right)}M_{jj'}-M_{..}=g_{j}+\left({\bar{I}}_{j.}-{\bar{I}}_{..}\right)$ , and $s_{jj'}^{*}=s_{jj'}+\left(I_{jj^{\prime }}-{\bar{I}}_{j.}-{\bar{I}}_{j^{\prime }.}+2{\bar{I}}_{..}\right)$ , where $g_{j}$ and $s_{jj'}$ are the GCA and SCA effects assuming no epistasis. Note that $\sum_{j=1}^{N}g_{j}^{*}=0$ , because $\sum_{j=1}^{N}g_{j}=0$ , and $\sum_{j=1<}^{N-1}\sum_{j^{\prime }=2}^{N}s_{jj'}=0$ . However, $\sum_{j=1<}^{N-1}\sum_{j^{\prime }=2}^{N}s_{jj'}^{*}=\left[N\left(N-1\right)/2\right]{\bar{I}}_{..}$ because $\sum_{j=1<}^{N-1}\sum_{j^{\prime }=2}^{N}I_{jj^{\prime }}\neq 0$ .

### Simulation

The simulated dataset was generated using the software *REALbreeding* (available by request). *REALbreeding* has been used in studies related to genomic selection (Viana et al., 2019), GWAS (Pereira et al., 2018), QTL mapping (Viana et al., 2017), LD (Andrade et al., 2019), population structure (Viana et al., 2013b), heterotic grouping/genetic diversity (Viana et al., 2020), and plant breeding (Viana et al., 2013a). In summary, the software simulates individual genotypes for genes and molecular markers and phenotypes in three stages using inputs from user. The first stage (genome simulation) is the specification of the number of chromosomes, molecular markers, and genes as well as marker type and density. The second stage (population simulation) is the specification of the population(s) and sample size or progeny number and size. A population is characterized by the average frequency for the genes (biallelic) and markers (first allele). A beta distribution is employed to generate allele frequencies. The last stage (trait simulation) is the specification of the minimum and maximum genotypic values for homozygotes, the minimum and maximum phenotypic values (to avoid outliers), the direction and degree of dominance, and the broad sense heritability.

The minimum and maximum genotypic values for homozygotes are used to compute the a and d deviations. The current version allows the inclusion of digenic epistasis, genotype × environment interaction, and multiple traits (up to 10), including pleiotropy. The population mean (M) and additive (A), dominance (D), and epistatic (additive × additive (AA), additive × dominance (AD), dominance × additive (DA), and dominance × dominance (DD)) genetic values or general combining ability (GCA), specific combining ability (SCA), and epistatic (I) effects, or genotypic values (G), depending on the population, are calculated from the parametric gene effects and frequencies and the parametric LD values. The population in LD is generated by crossing two populations in linkage equilibrium followed by a generation of random cross. The parametric LD is $\Delta_{ab}^{\left(-1\right)}=\left[\left(1-2r_{ab}\right)/4\right]\left(p_{a1}-p_{a2}\right)\left(p_{b1}-p_{b2}\right)$ , where $r_{ab}$ is the recombination frequency, p is an allelic frequency, and the indexes 1 and 2 indicate the parental populations. The phenotypic values ( P ) are computed assuming error effects (E) sampled from a normal distribution ( P=M+A+D+AA+AD+DA+DD+ E=G+E or P=M+GCA1+GCA2+SCA+I+E=G+E ).

### Heterosis and combining ability analyses of populations

**Figure 1:**
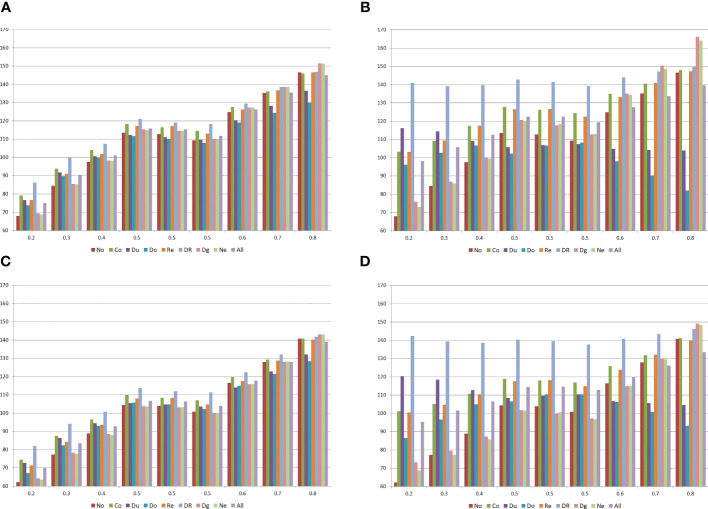
Means of the populations **(A, B)** and the selfed populations **(C, D)** for grain yield (g/plant), assuming no epistasis (No), seven types of digenic epistasis and an admixture of these types (All), 25% **(A, C)** and 100% **(B, D)** of epistatic genes, and ratio V(I)/(V(A) + V(D)) of 1. The populations are identified by the average allele frequency. Co = complementary, Du = duplicate, Do = dominant, Re = recessive, DR = dominant and recessive, Dg = duplicate genes with cumulative effects, and Ne = non-epistatic genic interaction.

Aiming to assess the impact of epistasis in the heterosis and combining ability analyses of populations, I simulated nine populations, the selfed populations, and the 36 interpopulation crosses (see the characterization of the populations in **Figure 1**), assuming 400 genes on 10 chromosomes of 200 cM (40 genes by chromosome) determining grain yield. The populations with average allelic frequency of 0.5 differ for the LD level (higher for population 4 and lower for population 6). I assumed positive dominance and average degree of dominance of 0.6 (range 0.1 to 1.2). The minimum and maximum genotypic values for homozygotes were 30 and 160 g/plant, respectively. The minimum and maximum phenotypic values were 10 and 180 g/plant, respectively. The broad sense heritability at the plant level was 10%, and the sample size was 100. I defined seven types of digenic epistasis and an admixture of these types, assuming 25 and 100% of epistatic genes.

The types of digenic epistasis are as follows: complementary ( $G_{22}=G_{21}=G_{12}=G_{11}$ and $G_{20}=G_{10}=G_{02}=G_{01}=G_{00}$ ; proportion of 9:7 in a F_2_, assuming independent assortment), duplicate ( $G_{22}=G_{21}=G_{20}=G_{12}=G_{11}=G_{10}=G_{02}=G_{01}$ ; proportion of 15:1 in a F_2_, assuming independent assortment), dominant ( $G_{22}=G_{21}=G_{20}=G_{12}=G_{11}=G_{10}$ and $G_{02}=G_{01}$ ; proportion of 12:3:1 in a F_2_, assuming independent assortment), recessive ( $G_{22}=G_{21}=G_{12}=G_{11}$ , $G_{02}=G_{01}$ , and $G_{20}=G_{10}=G_{00}$ ; proportion of 9:3:4 in a F_2_, assuming independent assortment), dominant and recessive ( $G_{22}=G_{21}=G_{12}=G_{11}=G_{20}=G_{10}=G_{00}$ and $G_{02}=G_{01}$ ; proportion of 13:3 in a F_2_, assuming independent assortment), duplicate genes with cumulative effects ( $G_{22}=G_{21}=G_{12}=G_{11}$ , and $G_{20}=G_{10}=G_{02}=G_{01}$ ; proportion of 9:6:1 in a F_2_, assuming independent assortment), and non-epistatic genic interaction ( $G_{22}=G_{21}=G_{12}=G_{11}$ , $G_{20}=G_{10}$ , and $G_{02}=G_{01}$ ; proportion of 9:3:3:1 in a F_2_, assuming independent assortment).

Because the genotypic values for any two interacting genes are not known, there are infinite genotypic values that satisfy the specifications of each type of digenic epistasis. For example, fixing the gene frequencies (the population) and the parameters m, a, d, and d/a (degree of dominance) for each gene (the trait), the solutions $G_{22}=G_{21}=G_{12}=G_{11}=5.25$ and $G_{20}=G_{10}=G_{02}=G_{01}=G_{00}=5.71$ or $G_{22}=G_{21}=G_{12}=G_{11}=6.75$ and $G_{20}=G_{10}=G_{02}=G_{01}=G_{00}=2.71$ define complementary epistasis but the genotypic values are not the same. The software allows the user to control the magnitude of the epistatic variance (V(I)), relative to the magnitudes of the additive and dominance variances (V(A) and V(D)). As an input for the user, the software requires the ratio V(I)/(V(A) + V(D)) for each pair of interacting genes (a single value; for example, 1.0). Then, for each pair of interacting genes the software samples a random value for the epistatic value $I_{22}$ (the epistatic value for the genotype AABB), assuming $I_{22}∼N\left(0,\;V\left(I\right)\right)$ . Then, the other epistatic effects and genotypic values are computed. I assumed ratios 1 and 10. Increasing the ratio increases the magnitude of the additive, dominance, and epistatic genetic values.

The influence of epistasis in the heterosis and combining ability analyses of the populations was measured by:

1. the correlations between the average frequency for the genes that increase the trait expression and the parametric (true) variety and GCA effects.
2. the correlations between the average absolute allelic frequency differences between populations and the parametric heterosis, specific heterosis, and SCA effect.
3. the correlations between the absolute allelic frequency differences between a population and the other diallel parents and the parametric variety heterosis and the absolute SCA effect of a population with itself.
4. the correlation between the absolute value of the average frequency for the genes that increase the trait expression minus 0.5 and the parametric change in the population mean due to inbreeding.

### Combining ability analysis of DHs

To assess the influence of epistasis in the combining ability analyses of DH lines, I used *REALbreeding* to sample 20 DHs from each population and to generate the 16,110 single crosses. The broad sense heritability for the DHs and single crosses were 30 and 70%, respectively. Again, because *REALbreeding* provides the genotype and the parametric genotypic value for each DH and the parametric values of the GCA, SCA, and epistatic effects for each single cross, I did not process the phenotypic data for their estimation. The impact of epistasis in the combining ability analyses of the DHs was measured by the correlations between the average frequency for the genes that increase the trait expression and the parametric GCA effect and between the average absolute allelic frequency differences and the parametric SCA effect. I also processed analyses sampling 20 DHs (from 180), which was replicated 100 times. To avoid the influence of the experimental error, experimental method 4, model I (Griffing, 1956), was fitted using the parametric single cross genotypic values.

## Results

Compared with the absence of epistasis, the existence of interallelic interactions can lead to an increase or decrease in the population mean and in the inbreeding depression, depending on the population allelic frequencies, type of epistasis, percentage of interacting genes, and ratio V(I)/(V(A) + V(D)) (not shown) (**Figure 1**). In general, the change (in absolute value) in the population mean was lower with duplicate epistasis and higher with dominant and recessive epistasis. The inbreeding depression was higher with duplicate genes with cumulative effects and non-epistatic genic interaction and lower with duplicate and dominant epistasis.

**Figure 2:**
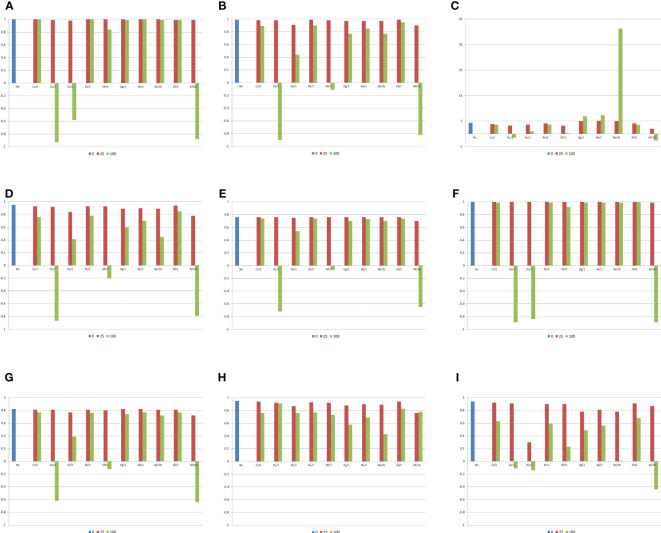
Correlations between the average frequency for the genes that increase the trait expression, the average absolute allelic frequency differences between populations, the absolute average allelic frequency differences between a population and the other diallel parents, or the average frequency for the genes that increase the trait expression minus 0.5 and the genetic components of the heterosis and combining ability analyses, and average heterosis (g/plant), assuming no epistasis (No), seven types of digenic epistasis and an admixture of these types (All), 25% and 100% of epistatic genes, and ratios V(I)/(V(A) + V(D)) of 1 and 10. **(A)** $v_{j}^{*}$ , **(B)** $H_{jj'}^{*}$ , **(C)** $H^{*}$ , **(D)** $H_{j}^{*}$ , **(E)** $S_{jj'}^{*}$ , **(F)** $g_{j}^{*}$ , **(G)** $s_{jj'}^{*}$ , **(H)** $s_{jj}^{*}$ , and **(I)** $d_{j}^{*}$ . b = no dominance.

If there is no epistasis, the heterosis and combining ability analyses of populations perfectly indicate the superior populations, from the estimates of the population means (unrestricted model), variety effects (restricted model), or GCA effects, and the most divergent populations, from the analysis of the heteroses (unrestricted model), heteroses effects (restricted model), or SCA effects (**Figure 2**). If there is epistasis, however, but depending on the predominant epistasis type, the percentage of interacting genes, and the magnitude of their effects, both analyses can lead to completely wrong inferences regarding the identification of the superior populations, the populations with greater differences of gene frequencies, and the populations with maximum variability (allelic frequencies close to 0.5). This will occur because of negative or lower correlations between variety mean/variety effect or GCA effect with the average allelic frequency, between heterosis/heterosis effect or SCA effect with the average allelic frequency difference, and between the change in the population mean due to inbreeding and the average frequency minus 0.5. This negative impact of epistasis on the heterosis and combining ability analyses will occur with duplicate, dominant, and dominant and recessive epistasis with 100% of epistatic genes, regardless of the ratio epistatic variance/(additive + dominance variances), that is, independent of the magnitude of the epistatic effects. Assuming a ratio of 10, the negative effect also occurs with an admixture of epistasis types.

Concerning the average heterosis, there are significant differences between the values observed assuming no epistasis (4.3 g/plant) and digenic epistasis (−2.6 to 7.3 g/plant), if there is dominance, proportional to the percentage of the interacting genes and magnitude of their effects. Excepting complementary, duplicate genes with cumulative effects, and non-epistatic genic interaction, assuming dominance there was a decrease in the average heterosis by increasing the percentage of epistatic genes and the magnitude of their effects. Note that, assuming no dominance, the increase in the percentage of the interacting genes increased the average heterosis under non-epistatic gene interaction (highest average heterosis). The influence of epistasis on both the variety and specific heterosis follows the effect described for heterosis. Interestingly, epistasis has a less pronounced effect on the SCA effect of a population with itself, compared with the effect observed on the change in the population mean due to inbreeding.

**Figure 3:**
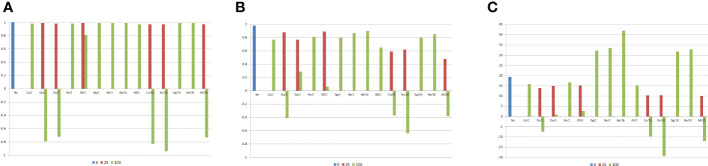
Correlations between the average frequency for the genes that increase the trait expression or the average allelic frequency differences between the DH lines and the genetic components of the combining ability analysis, and average heterosis (g/plant), assuming no epistasis (No), seven types of digenic epistasis and an admixture of these types (All), 25% and 100% of epistatic genes, ratio V(I)/(V(A) + V(D)) of 1 and 10, and 20 DHs by population. **(A)** $g_{j}^{*}$ , **(B)** $s_{jj'}^{*}$ , and **(C)** $H^{*}$ . b = no dominance.

**Figure 4:**
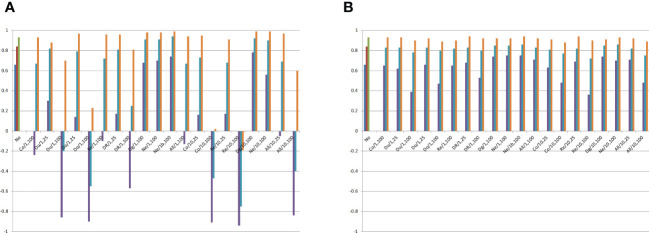
Minimum, average, and maximum correlations between the average frequency for the genes that increase the trait expression or the average allelic frequency differences between the DH lines and the genetic components of the combining ability analysis, assuming no epistasis (No), seven types of digenic epistasis and an admixture of these types (All), 25% and 100% of epistatic genes, ratio V(I)/(V(A) + V(D)) of 1 and 10, and 100 samples of 20 DHs. **(A)** $g_{j}^{*}$ and **(B)** $s_{jj'}^{*}$ . Co = complementary, Du = duplicate, Do = dominant, Re = recessive, DR = dominant and recessive, Dg = duplicate genes with cumulative effects, and Ne = non-epistatic genic interaction. b = no dominance.

The previous results were in general also observed for the combining ability analysis of all 180 DH lines (**Figures 3**, **4**), that is, a negative impact of epistasis on the identification of the superior and the most contrasting DHs assuming duplicate and dominant epistasis with 100% of interacting genes, regardless of the ratio V(I)/(V(A) + V(D)). There was also a negative influence of complementary and recessive epistasis, as well as of an admixture of epistasis types under a ratio of 10. No impact on the combining ability analysis of DHs was observed for duplicate genes with cumulative effects and non-epistatic genic interaction, even assuming 100% of interacting genes and ratio 10 (**Figure 3**). Regardless of the ratio V(I)/(V(A) + V(D)), there was maximization of the average heterosis with duplicate genes with cumulative effects (32.2 g/plant) and non-epistatic genic interaction (33.5 g/plant). For the other epistasis types and admixture of epistasis types, increasing the percentage of epistatic genes and the ratio V(I)/(V(A) + V(D)) decreased the average heterosis.

Surprisingly, the combining ability analyses of 100 subsets of 20 DHs showed no significant negative average impact of epistasis on the identification of the most divergent DHs, even assuming 100% of epistatic genes and ratio of 10 (**Figure 4**). The minimum correlation between SCA effect and the average allelic frequency difference was 0.66 under no epistasis and 0.36 assuming recessive epistasis, 100% of epistatic genes, and ratio of 10. Only with duplicate genes with cumulative effects, non-epistatic genic interaction, and dominant and recessive epistasis, under 100% of epistatic genes, can epistasis negatively affect the identification of the superior DHs even assuming a ratio V(I)/(V(A) + V(D)) of 1. By increasing the ratio to 10, the same negative influence of epistasis occurred for complementary and recessive epistasis, as well as for an admixture of epistasis types.

## Discussion

Based on a huge amount of empirical data, geneticists agree that genotypic value is mainly attributable to additive effects of genes and intra-allelic interactions (dominance). Reviewing empirical data, especially results from QTL mapping, Mackay (2014) emphasizes that epistasis is common for quantitative traits but with a controversial significance. For the author, the controversial role of epistasis is simply because inter-allelic interactions are more difficult to detect. In recent investigations based on transcriptome analysis and genomic prediction of complex traits, epistasis was observed (Vitezica et al., 2018; Zhao et al., 2019). Based on the available quantitative genetics theory (Minvielle, 1987; Schnell and Cockerham, 1992; Cockerham and Zeng, 1996; Kao and Zeng, 2002; Garcia et al., 2008), geneticists also agree that epistasis can determine heterosis but with a controversial role. However, the controversial significance of epistasis on heterosis is simply because it is difficult to measure the relative importance of intra- and interallelic interaction. Nevertheless, it should be emphasized that most of the empirical results indicate a higher significance of dominance (Garcia et al., 2008; Kaeppler, 2012; Schnable and Springer, 2013; Liu et al., 2020b; Mackay et al., 2021). However, in agreement with our results, Jiang et al. (2017) observed that dominance effects contributed less than epistatic effects to wheat grain-yield heterosis.

Most of the empirical evidence for epistasis came from QTL mapping (Mackay, 2014). However, QTL mapping provides limited estimates of genetic effects and degree of dominance since they refer only to identified QTLs. Schnell and Cockerham (1992) emphasize that the marker contrasts estimate only a small fraction of epistatic effects for linked QTLs. Furthermore, the estimates for low heritability QTLs show high sampling error (Viana et al., 2017). Due to missing heritability, genomic prediction also provides limited estimates of genetic variances (no one covariance) (Kim et al., 2017). Thus, geneticists agree that a significant contribution to the knowledge on the role of epistasis in determining quantitative traits and their genetic variability should come from the analysis of theoretical models and from simulated data generated based on the theoretical models (Maki-Tanila and Hill, 2014; Hill and Maki-Tanila, 2015).

The quantitative genetics theory presented in this study reveals several important new findings, confirm a previous inference from Minvielle (1987) (under LD and epistasis, there is heterosis without dominance), and clearly show that the breeders cannot test epistasis when processing heterosis and combining ability analyses of populations or DH/inbred/pure lines. The highlights are as follows: 1) the epistatic linear components of populations, selfed populations, and interpopulation crosses cannot be estimated because they cannot be separated from the variety and heterosis components; 2) the parametric epistatic effects of the parents (populations or pure/inbred/DH lines) and F1s of a diallel cannot be estimated because they cannot be separated from the GCA and SCA components; 3) the heterosis and combining ability analyses do not allow testing epistasis; 4) epistasis determines panmitic population means only under LD; thus, epistasis determines heterosis only under LD; 5) only additive × additive and dominance × dominance effects affect heterosis between panmitic populations; 6) all epistatic effects affect the change in the population mean due to inbreeding (inbreeding depression), and thus, all epistatic effects affect the heterosis involving inbred populations; 7) in a combining ability analysis of populations, only additive × additive and dominance × dominance effects affect the GCA and SCA effects; and 8) in a combining ability analysis of pure/inbred/DH lines, all epistatic effects affect the GCA and SCA effects.

The results from the analyses of the simulated data demonstrate that epistasis can have a negative impact on the heterosis and combining ability analysis of panmitic populations, leading to wrong inferences regarding the identification of superior and most divergent populations. However, this depends on the type of predominant epistasis, percentage of epistatic genes, and magnitude of the epistatic effects. We observed a negative impact with duplicate, dominant, and dominant and recessive epistasis, assuming 100% of epistatic genes and a value 1 for the ratio epistatic variance/(additive plus dominance variances), and under an admixture of epistasis types and ratio 10 (high magnitude of the epistatic effects). In general, there was a decrease in the average heterosis by increasing the percentage of epistatic genes and the magnitude of their effects. A negative impact of epistasis on the identification of the superior and the most contrasting DHs was observed assuming duplicate and dominant epistasis with 100% of interacting genes, regardless of the magnitude of the epistatic effects. There was also a negative influence of complementary and recessive epistasis, as well as of an admixture of epistasis types under a value of 10 for the ratio epistatic variance/(additive plus dominance variances). Regardless of the ratio, there was maximization of the average heterosis with duplicate genes with cumulative effects and non-epistatic genic interaction. For the other epistasis types and admixture of epistasis types, increasing the percentage of epistatic genes and the ratio epistatic variance/(additive plus dominance variances) decreased the average heterosis. Surprisingly, the combining ability analyses of 100 subsets of 20 DHs showed no significant average negative impact of epistasis on the identification of the most divergent DHs, even assuming 100% of epistatic genes and ratio of 10. Our analysis assuming no dominance showed that the magnitude of the average heterosis can significantly increase, as exemplified assuming a non-epistatic genic interaction, 100% of epistatic genes, and a ratio of 1. For populations and DHs, the average heterosis achieved impressive values (44% and 58% respectively, relative to parents mean).

As previously emphasized, breeders cannot even test epistasis in the heterosis and combining ability analyses simply because there is a distinct epistatic component of mean for each population, selfed population, DH/inbred/pure line, and their F_1_. Thus, it is not possible to estimate these epistatic components. This finding implies that breeders cannot avoid the negative impact of epistasis in the heterosis and combining ability analyses if the genetic system involves a high number of epistatic genes with great effects. Concerning the relative magnitude of the epistatic genetic values, I observed that, when the impact of epistasis was negative, not necessarily the absolute value of the epistatic values was superior to the absolute value of the additive value. For DHs, assuming duplicate epistasis, 100% of epistatic genes, and ratio V(I)/(V(A) + V(D)) of 1, the absolute epistatic value corresponded to 13%, on average, of the single cross genotypic value.

In conclusion, I have a positive message for the breeders: in general, especially if only a minor fraction of the genes are epistatic or if the magnitude of the epistatic effects are of reduced magnitude, the epistasis will not have any impact on the heterosis and combining ability analyses. However, breeders should be conscious that a negative impact can occur. I also emphasize that our simulated data provided results that are supported from field data, for example, higher heterosis for the most contrasting populations that can be assumed as heterotic groups, e.g., 1.4% and 10.5% for the heterosis involving populations 1×2 and 1×10, respectively, assuming a ratio of 1, 100% of epistatic genes, and an admixture of epistasis types; higher heterosis for interpopulation single crosses relative to the intrapopulation heterosis, for example, average intra- and interpopulation heteroses of 12.0% and 15.6%, also assuming a ratio of 1, 100% of epistatic genes, and an admixture of epistasis types; and finally, lower percent values of the average heterosis for populations (in the range −2.1 to 6.2) than for DHs (in the range −12.2 to 36.6), as observed in several studies (Laude and Carena, 2015; Jiang et al., 2017; Lariepe et al., 2017; Punya et al., 2019; Yu et al., 2020).

## Data availability statement 

The datasets presented in this study can be found in online repositories. The names of the repository/repositories and accession number(s) can be found at: 10.6084/m9.figshare.14944608.

## Author contributions 

The author confirms being the sole contributor of this work and has approved it for publication.

## Appendix

The epistatic components of the genotypic mean for the interpopulation cross between populations j and j′ can be derived from the gametic probabilities and epistatic effects of the genotypic values (G) summarized in the table below, where the genotypic values are defined by Kempthorne (1954),

**Table:**

	$p_{aj'}p_{bj'}+\Delta_{abj'}^{\left(0\right)}$	$p_{aj'}q_{bj'}-\Delta_{abj'}^{\left(0\right)}$	$q_{aj'}p_{bj'}-\Delta_{abj'}^{\left(0\right)}$	$q_{aj'}q_{bj'}+\Delta_{abj'}^{\left(0\right)}$	
$p_{aj}p_{bj}+\Delta_{abj}^{\left(0\right)}$	$G_{22}$	$G_{21}$	$G_{12}$	$G_{11}$	$E{\left(G\right)}_{11j}$
$p_{aj}q_{bj}-\Delta_{abj}^{\left(0\right)}$	$G_{21}$	$G_{20}$	$G_{11}$	$G_{10}$	$E{\left(G\right)}_{10j}$
$q_{aj}p_{bj}-\Delta_{abj}^{\left(0\right)}$	$G_{12}$	$G_{11}$	$G_{02}$	$G_{01}$	$E{\left(G\right)}_{01j}$
$q_{aj}q_{bj}+\Delta_{abj}^{\left(0\right)}$	$G_{11}$	$G_{10}$	$G_{01}$	$G_{00}$	$E{\left(G\right)}_{00j}$
	$E{\left(G\right)}_{11j'}$	$E{\left(G\right)}_{10j'}$	$E{\left(G\right)}_{01j'}$	$E{\left(G\right)}_{00j'}$	$E{\left(G\right)}_{jj'}$

For example, assuming the restrictions defined by Kempthorne (1954), the marginal means for the additive × additive effects, fixing a population, are

$$E{\left(AA\right)}_{11j}=\left(p_{aj'}p_{bj'}+\Delta_{abj'}^{\left(0\right)}\right)\left(4\alpha_{A}\alpha_{B}\right)+\ldots +\left(q_{aj'}q_{bj'}+\Delta_{abj'}^{\left(0\right)}\right)\left(\alpha_{A}\alpha_{B}+\alpha_{A}\alpha_{b}+\alpha_{a}\alpha_{B}+\alpha_{a}\alpha_{b}\right)=\alpha_{A}\alpha_{B}+\Delta_{abj'}^{\left(0\right)}\left(aa\right)$$

E(AA)10j=αAαb+Δabj'(0)(aa)

E(AA)01j=αaαB+Δabj'(0)(aa)

E(AA)00j=αaαb+Δabj'(0)(aa)

Then,

$$E{\left(AA\right)}_{jj'}=\Delta_{abj}^{\left(0\right)}\left(aa\right)+\Delta_{abj'}^{\left(0\right)}\left(aa\right)$$
